# Differential Regulation of Glutamate Transporter Subtypes by Pro-Inflammatory Cytokine TNF-α in Cortical Astrocytes from a Rat Model of Amyotrophic Lateral Sclerosis

**DOI:** 10.1371/journal.pone.0097649

**Published:** 2014-05-16

**Authors:** Amélie O. Dumont, Stéphanie Goursaud, Nathalie Desmet, Emmanuel Hermans

**Affiliations:** Institute of Neuroscience, Group of Neuropharmacology, Université Catholique de Louvain, Brussels, Belgium; Inserm, France

## Abstract

Dysregulation of the astroglial glutamate transporters GLAST and GLT-1 has been implicated in several neurodegenerative disorders, including amyotrophic lateral sclerosis (ALS) where a loss of GLT-1 protein expression and activity is reported. Furthermore, the two principal C-terminal splice variants of GLT-1 (namely GLT-1a and GLT-1b) show altered expression ratio in animal models of this disease. Considering the putative link between inflammation and excitotoxicity, we have here characterized the influence of TNF-α on glutamate transporters in cerebral cortical astrocyte cultures from wild-type rats and from a rat model of ALS (hSOD1^G93A^). Contrasting with the down-regulation of GLAST, a 72 h treatment with TNF-α substantially increased the expression of GLT-1a and GLT-1b in both astrocyte cultures. However, as the basal level of GLT-1a appeared considerably lower in hSOD1^G93A^ astrocytes, its up-regulation by TNF-α was insufficient to recapitulate the expression observed in wild-type astrocytes. Also the glutamate uptake activity after TNF-α treatment was lower for hSOD1^G93A^ astrocytes as compared to wild-type astrocytes. In the presence of the protein synthesis inhibitor cycloheximide, TNF-α did not influence GLT-1 isoform expression, suggesting an active role of dynamically regulated protein partners in the adaptation of astrocytes to the inflammatory environment. Confirming the influence of inflammation on the control of glutamate transmission by astrocytes, these results shed light on the regulation of glutamate transporter isoforms in neurodegenerative disorders.

## Introduction

Disturbances in glutamate homeostasis, which lead to toxic accumulation of this excitatory neurotransmitter in the synaptic cleft, are observed in several neuropathologies. Indeed, excessive activation of ionotropic glutamate receptors is commonly associated with irreversible excitotoxic damages to neuronal cells [1]. Specific high-affinity transporters contribute to the rapid and efficient clearance of glutamate by which the nervous parenchyma is protected against the extracellular accumulation of glutamate. Among the five subtypes of glutamate transporters identified in cells of the nervous parenchyma, the astroglial glutamate transporters GLAST (the rodent homologue of human EAAT1) and GLT-1 (EAAT2) [2] are best characterized as they likely ensure most of the glutamate uptake in physiological conditions. Hence, dysfunction of these two glutamate transporters is clearly implicated in neurodegenerative insults, in particular amyotrophic lateral sclerosis (ALS) [3], [4], a fatal disease characterized by the progressive and specific loss of motor neurons that has been correlated with deficient GLT-1 expression and activity [5], [6]. The subsequent identification of splice variants of this transporter was followed by the demonstration of distinct regulatory profiles for the most abundant isoforms, namely GLT-1a and GLT-1b [7], [8]. Differences in the regulation of these splice variants could indeed explain their altered expression ratio in pathological contexts, including ALS [9]–[11]. It is, however, noteworthy that so far, the majority of biochemical studies failed to distinguish between these two isoforms or essentially focused on GLT-1a because the distinctive roles of each of these isoforms and their physiological relevance remain poorly understood.

A key pathological feature of ALS in both human and animal models is the extensive astrocytic activation and microgliosis which accompanies the release of inflammatory mediators [12], [13]. Hence, excitotoxicity and neuroinflammation are concomitant in various neuropathologies such as Parkinson disease, multiple sclerosis, Alzheimer disease or ALS [3], [14]. Indeed, the expression and activity of glutamate transporters are influenced by cytokines, growth factors and reactive oxygen species (ROS), common mediators of neuroinflammation [15]. In this study, we aimed at comparing the influence of an inflammatory environment on the glutamate transporters GLAST, GLT-1a and GLT-1b, in primary cultures of cortical astrocytes derived from wild-type or transgenic rats carrying a mutated form of the human superoxide dismutase 1 (hSOD1^G93A^). These rodents recapitulate the symptoms and biochemical features of ALS and the strain constitutes a validated model of the disease even though the hSOD1^G93A^ expression was so far never validated in primary cultures of astrocytes. Mimicking the inflammation detected in the nervous tissues of ALS rats [16], [17], the cell cultures were exposed to tumor necrosis factor α (TNF-α), a pro-inflammatory cytokine for which glial cells from this transgenic rat strain show increased immune reactivity [16], [18].

## Materials and Methods

### Animals and Ethics Statement

All experiments were conducted on samples collected from Sprague-Dawley rats maintained in strict adherence to the EU directive of 22/09/2010 (2010/63/EU). The ethical committee of the Université catholique de Louvain for animal experiments specifically approved this study, which received the agreement nu 2010/UCL/MD/032. Our laboratory received agreement number LA 1230297 from the Ministry of Agriculture. All animals were housed in cages that provided an environment with controlled light/dark cycles, temperature and humidity. Every effort was made to minimize suffering during the manipulations and rats were euthanized with CO2, except for pups that were sacrificed by decapitation. Rats carrying the hSOD1^G93A^ transgene were initially obtained from Dr. R. Pochet (Université Libre de Bruxelles, Belgium). These rats were genotyped after birth with genomic DNA extracted from a tail biopsy using PCR as previously described [11].

### Tissue Dissection

To measure the expression of the endogenous (rSOD1) and exogenous (hSOD1) SOD1 in mutant rats, wild-type and hSOD1^G93A^ males were sacrificed at various stages of the disease to collect the lumbar spinal cord or the cortex. The weight and the motor score of each animal developing ALS disease were evaluated as previously described by Matsumoto [19]. At various stages of the disease (at postnatal day (P) 5, the non-paralysed stages P60 and P120, first signs of paralysis P150, and end-stage of the disease P195 corresponding to the motor score 0), rats were euthanized with CO_2_ (except at P5; here decapitation was used) and the lumbar spinal cord was collected and the meninges were removed. Tissues were immediately frozen in liquid nitrogen and stored at −80°C until use.

### Astroglial Culture and Treatments

Primary cultures from the cortex of either wild-type or transgenic hSOD1^G93A^ rats pups (P4–5) were performed immediately after genotyping. Cerebral cortices of these pups were collected and meninges were carefully removed and kept in Petri dishes containing a solution of PBS/2% glucose. Tissues were then suspended in PBS/glucose supplemented with 10 mg/mL trypsin (Worthington, United States), 1 mg/mL DNase (Worthington), 5 mg/mL MgSO_4_ (Sigma-Aldrich, Diegem, Belgium) at 37°C for 3 min and washed with PBS/glucose. Tissues were mechanically dissociated in Dulbecco’s Modified Eagle Medium (DMEM, Life Technologies, Gent, Belgium) containing 0.5 mg/mL DNase. After a centrifugation of 5 min at 1200 *g*, cells were suspended in DMEM containing 4.5 g/L glucose, 25 mmol/L HEPES and supplemented with 10% fetal bovine serum (FBS, Thermoscientific, Tournai, Belgium), 100 units/mL penicillin, 100 mg/mL streptomycin and 6 mmol/L glutamine (Life Technologies) and aggregates were removed by filtration using a strainer with a pore size of 100 µm. Isolated cells were then incubated for growing at 37°C in a humidified atmosphere containing 5% CO_2_ into 175 cm^2^ culture flasks and culture medium was renewed after one week. Non astroglial cells such as microglia and oligodendrocytes were removed after 10 days by 24 h shaking at 200 rpm on an orbital shaker. After two weeks of proliferation, cells were collected by trypsinisation (trypsin-EDTA 0.05%, Life Technologies) and plated at 10^4^ cells/cm^2^. Cells were seeded into non-coated 6-well plates for quantitative PCR measurements and Western blotting, or were distributed on poly-L-lysine coated 12-well plates for d-[^3^H]-aspartate uptake measurement. After 48 h, cells were fed with new culture medium containing 3% of FBS during 4 days. Then, astrocyte treatment with TNF-α (20 ng/mL, AbD Serotec, Oxford, England) in the same medium was commenced and lasted 72 h; alternatively, astrocytes were incubated for 48 h with cycloheximide (10 µg/mL, Sigma Aldrich), supplemented or not with 20 ng/mL TNF-α.

### RNA Extraction, Reverse Transcription and Quantitative PCR Measurements

Total RNA extraction from cells was carried out using the Tripure Isolation Reagent (Roche Diagnostic, Mannheim Germany) following the manufacturer’s protocol. Samples were treated with RQ1 RNase-free DNase kit (Promega Benelux, Leiden, The Netherlands) to remove DNA contamination before cDNA synthesis. Reverse transcription and quantitative PCR were performed as previously detailed [11]. Amplification of each target transcript with appropriates primers (Table 1, Life Technologies) was performed in the same conditions with serial dilutions of a cloned fragment of the corresponding transporter cDNA sequence. Each sample was normalized with the relative expression of GAPDH and TATA binding protein (TBP). Thereby, quantification of GLT-1a, GLT-1b and GLAST was carried out using plasmids expressing one or the other transcript as internal control and reported as cDNA copy number per µg of total RNA.

**Table 1:** PCR primers (F : forward primer, R : reverse primer) and size of amplicon.

Gene	Sequence	Amplicon (bp)
GAPDH	F : 5′-GTCTCCTGTGACTTCAACAG-3′	76
	R : 5′-AGTTGTCATTGAGAGCAATGC-3′	
TBP	F : 5′-CAGGAGCCAAGAGTGAAGAAC-3′	251
	R : 5′-AGGAAATAATTCTGGCTCATAGCTACT-3′	
GLT-1a	F : 5′-TGTCTATGCCGCACACAACT-3′	90
	R : 5′-TCCTCAACACTGCAGTCAGC-3′	
GLT-1b	F : 5′-AATGTGTCTATGCCGCACAC-3′	128
	R : 5′-GCAGGGGATGGTGCTTTT-3′	
GLAST	F : 5′-GGATGGAAAGATTCCAGCAA-3′	128
	R : 5′-GCTGACGGTGAGTAGCACAA-3′	

### Western Blotting

Astrocytes seeded in 6-well plates were rinsed with PBS and scraped in ice-cold lysis buffer (Tris 10 mM, pH 7.4, ethylene diamine tetraacetic acid 1 mM, ethylene glycol tetraacetic acid 10 mM, DL-dithiotreitol (DTT) 2 mM, Igepal-NP40 1%, glycerol 20%) while lumbar spinal cord and cortex tissue previously dissected were homogenized in the same lysis buffer using a prechilled Teflon/glass homogenizer. Samples were then centrifuged (1000 *g*, 3 min) to remove insoluble material and sonicated. Protein concentration was determined by Lowry method, using DC Protein Assay Reagents Package (BioRad Laboratories) and samples were diluted in loading buffer (Tris 125 mM pH 6.8, glycerol 20%, sodium dodecyl sulfate (SDS) 4% and bromophenol blue 0.01%) and boiled for 5 min. Samples were electrophoresed through a 10% or a 14% (for SOD1) SDS-PAGE and transferred to nitrocellulose membrane by electroblotting. Membrane were incubated 30 min in Tris-buffered saline (TBS-Tris 50 mM pH 7.4, NaCl 150 mM) containing 0.05% Tween-20 and 5% non-fat milk to reduce non-specific labeling. Immunoprobing was carried out by incubating membranes overnight at 4°C with primary antibodies recognizing GLT-1a (guinea pig polyclonal antibody used at 1∶2500, Millipore, Brussels, Belgium), GLT-1b (rat monoclonal antibody, 1∶1500, previously characterized in our laboratory [7]), GLAST (goat polyclonal antibody, 1∶3000, Santa Cruz Biotechnology, Heildelberg, Germany) hSOD1/rSOD1 (rabbit polyclonal antibody, 1∶2000, LF-PA0013, AbFrontier - Gentaur Molecular Products BVBA, Belgium) and GAPDH (rabbit polyclonal antibody, 1∶30 000, Sigma Aldrich). Membrane were then incubated 1 h at 22°C with peroxidase-conjugated secondary antibody, goat anti-guinea pig IgG (1∶5000, Sigma), mouse monoclonal anti-rat IgG (1∶4000, Laboratory of Experimental Surgery, UCL, Belgium), rabbit anti-goat IgG (1∶2000, Sigma) and goat anti-rabbit IgG (1∶3000, Sigma), respectively. Immunoreactive proteins were detected with enhanced chemiluminescence reagent (Perkin Elmer NEN, Zaventem, Belgium) followed by autoradiography. Densitometric analysis of the signal was performed using ImageJ (Broken Symmetry Software). Of note, while picture depicting the immunoblots were rearranged for display in some figures, samples from WT and hSOD1^G93A^ rats were systematically run on the same gel allowing for comparison of the immunoreactive signals.

### d-[^3^H]-aspartate Uptake Measurement

Glutamate transporters activity was evaluated by uptake assays using d-aspartate, a transportable analogue of l-glutamate, which is not metabolized and does not interact with glutamate receptors. At the end of treatment with TNF-α, plates were placed at the surface of a 37°C water bath. Cells were rinsed three times with Krebs buffer (25 mmol/L HEPES, 4.8 mmol/L KCl, 1.2 mmol/L KH_2_PO_4_, 1.3 mmol/L CaCl_2_, 1.2 mmol/L MgSO_4_, 6 mmol/L glucose and 140 mmol/L NaCl, pH 7.4). d-[^3^H]-aspartate (50 nmol/L, specific activity of 11.3 Ci/mmol, Perkin Elmer) was added on the cells in presence or not of the appropriate inhibitors of glutamate transporters. Inhibitors of GLT-1 (WAY-213613, Tocris, Bristol, UK) and GLAST (UCPH-101, Tocris) were used at 100 and 10 µmol/L, respectively. After 6 min, uptake was stopped by three rinses with cold Krebs buffer (NaCl replaced by choline chloride) and cells were lysed with NaOH 0.1 N. Radioactivity was measured using the liquid scintillation solution Microscint 40 and the TopCount NXT Microplate Scintillation and Luminescence Counter (Perkin Elmer). A fraction of the lysate was used for protein determination by Bradford method using BioRad Protein Assay Dye Reagent (BioRad). Glutamate uptake rate was expressed as picomole of d-[^3^H]-aspartate transported per minute and per milligram of protein.

### Statistical Analyses

Statistical analyses were performed with GraphPad Prism version 5.03 (GraphPad software, San Diego, CA, USA). Data were expressed as means with standard error of the mean (SEM) and results were analyzed by two-way ANOVA followed by a Bonferroni post-hoc test. Values of *p*<0.05 were considered as statistically significant.

## Results

### Expression of hSOD1 in Cultured Astrocytes from hSOD1^G93A^ Rats

**Figure 1:**
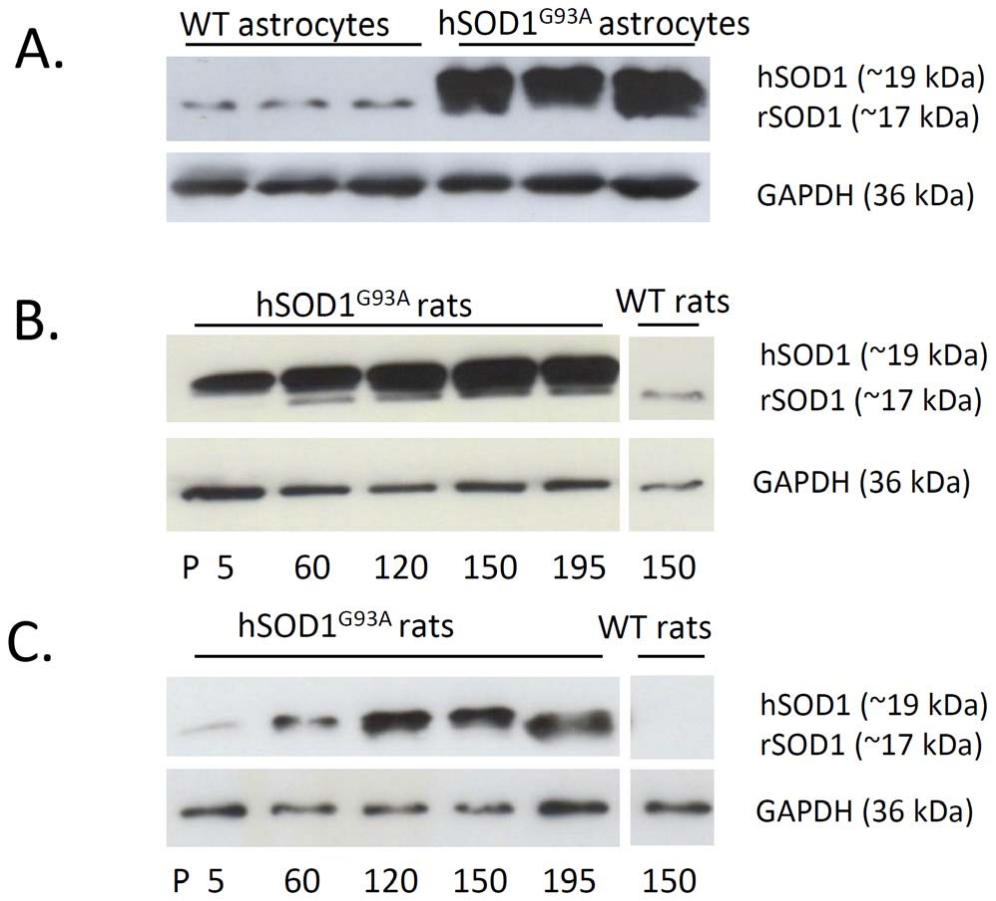
rSOD1 and hSOD1 protein expression in primary astrocytes cultures, lumbar spinal cord and cortex from wild-type and hSOD1^G93A^ rats. (A) Expression of the endogenous rSOD1 (around 17 kDa) and the exogenous hSOD1 (around 19 kDa) determined by Western blotting in wild-type or hSOD1^G93A^ astrocytes (samples from 3 different cultures are shown). The close molecular weight between the two SOD1 forms impairs clear-cut discrimination of rSOD1 when hSOD1 is expressed. Expression of the rSOD1 and hSOD1 proteins examined in the lumbar spinal cord (B) and cortex (C) from hSOD1^G93A^ rats at various stages of the disease (P5, P60, P120, P150 and P195) or from wild-type rats at P150. Immunoblots shown are representative of three independent experiments.

The ALS model overexpressing hSOD1^G93A^ is abundantly used to examine the molecular mechanisms underlying the disease. During aging, transgenic animals develop typical features of the disease and this is correlated with the progressive accumulation of the mutated SOD1 protein in the cytoplasm of both neurons and glial cells [20], [21]. While cortical astrocyte cultures derived from newborn rodents are widely used to study specific astrocytic alteration associated with diverse insults, the expression of the human mutated form of SOD1 has never been characterized in cultures derived from hSOD1^G93A^ rat pups. Herein, we therefore investigated the expression of SOD1 in cortical astrocytes. The human and rat SOD1 are recognized by the same antibody but they show different molecular weights and can be distinguished after SDS-PAGE and Western blotting. Our data shown in Figure 1A clearly evidence the expression of hSOD1 in cultured cortical astrocytes from hSOD1^G93A^ rodents. As expected, the rSOD1 protein was detected in astrocytes from both wild-type and transgenic rats, even though the signal corresponding to this endogenous protein is considerably lower as compared to the human protein in the hSOD1^G93A^ samples. These results appeared similar with those depicting the expression of SOD1 in the lumbar spinal cord of hSOD1^G93A^ rats in which a high expression of the hSOD1 is already detected at P5 (Fig. 1B). In the cortex, expression of the human protein was actually low at early stages, but increased with aging (Fig. 1C). This suggests that in vitro maturation of cortical astrocytes promotes the expression of the transgene and that these cells constitute a validated model to study the influence of hSOD1^G93A^ in astrocytes.

### TNF-α Down-regulates the mRNA and Protein Expression of GLAST in Wild-type and hSOD1^G93A^ Astrocytes

**Figure 2:**
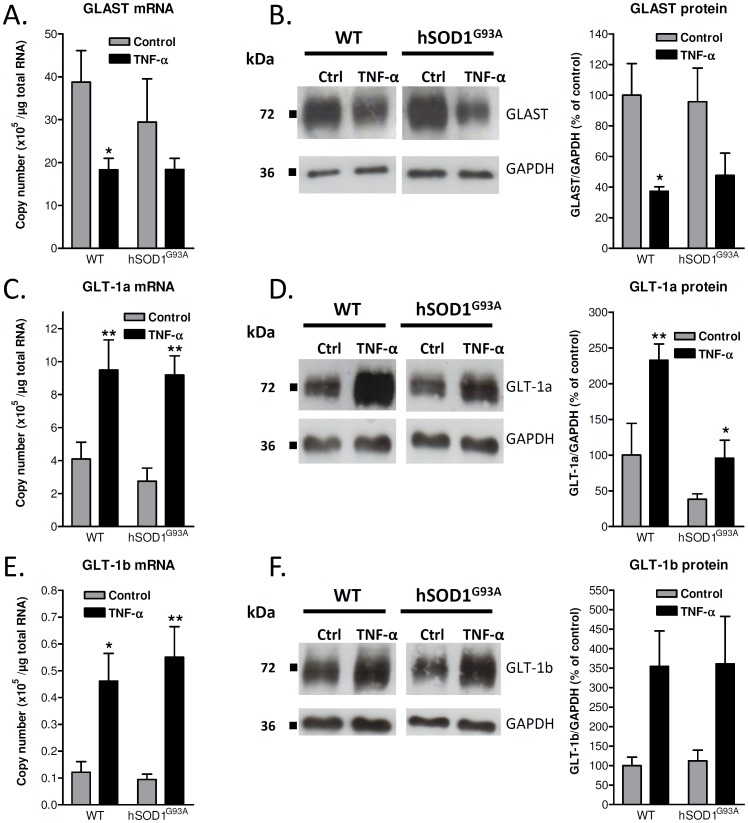
Influence of TNF-α on GLAST, GLT-1a and GLT-1b mRNAs and proteins in cortical astrocytes from wild-type or hSOD1^G93A^ rats. Expression of GLAST (A), GLT-1a (C) and GLT-1b (E) mRNAs as number of copies per microgram of RNA was estimated by RT-qPCR in control conditions or after 72 h exposure to TNF-α (20 ng/mL) using the corresponding cloned cDNA sequences as standards. Data shown are means with SEM conducted from six independent experiments performed in duplicate. Expression of GLAST (B), GLT-1a (D) and GLT-1b (F) proteins was examined in cells maintained in control conditions or treated with TNF-α (20 ng/mL) for 72 h. Immunoblots shown are representative of six independent experiments. Data indicate the levels of the protein of interest normalized to GAPDH and represents means with SEM. *p<0.05 for comparison between the same genotype.

In absence of treatment with TNF-α, the level of GLAST transcript tends to be lower in transgenic astrocytes as compared to wild-type cells, but we failed to measure any statistical significance for this observation. The addition of TNF-α for 72 h induced a clear down-regulation of the GLAST mRNAs in wild-type astrocytes, reaching a 50% decrease in comparison with the control condition and a trend of decrease in hSOD1^G93A^ cultures (Fig. 2A). Besides, the cytokine strongly reduced the level of GLAST proteins (at least 50% of reduction in comparison with control conditions) both in wild-type and ALS cultured astrocytes, correlating with the effects observed at the mRNA level (Fig. 2B).

### TNF-α Up-regulates the mRNA and Protein Expression of GLT-1a and GLT-1b Splice Variants

The influence of inflammatory stimuli on GLT-1 was previously examined both in vitro and in vivo, leading to controversies in the literature. We herein specifically investigated the effect of TNF-α on the transcriptional and protein expression of the principal C-terminus splice variants GLT-1a and GLT-1b in astrocyte cultures. At variance with the down-regulation observed for the GLAST, a 72 h treatment with TNF-α resulted in a substantial increase in the mRNA expression of GLT-1a (Fig. 2C). Even though the absolute mRNA copy number of GLT-1b appeared considerably lower than that of GLT-1a, the expression of the former was also robustly increased after exposure of the cultures to TNF-α (Fig. 2E). The up-regulation of GLT-1 isoforms induced by this cytokine was similar in astrocytes derived from wild-type animals and hSOD1^G93A^ rats (Fig. 2C & 2E). The up-regulation of GLT-1a mRNA by TNF-α was correlated with a 2.3-fold increase in the corresponding protein in wild-type astrocytes. In hSOD1^G93A^ astrocytes, the GLT-1a protein expression appeared lower than in wild-type cells, but the relative up-regulation observed after TNF-α treatment was similar (2.5-fold increase). This increase did, however, not recapitulate the expression observed in wild-type astrocytes in absence of treatment (Fig. 2D). The level of GLT-1b protein was similar in cultures from both genotypes and showed a comparable up-regulation after TNF-α treatment (3.5-fold increase), in correlation with the regulation of GLT-1b mRNA (Fig. 2F).

### Effect of an Inhibition of Protein Synthesis on the Expression of Glutamate Transporters

**Figure 3:**
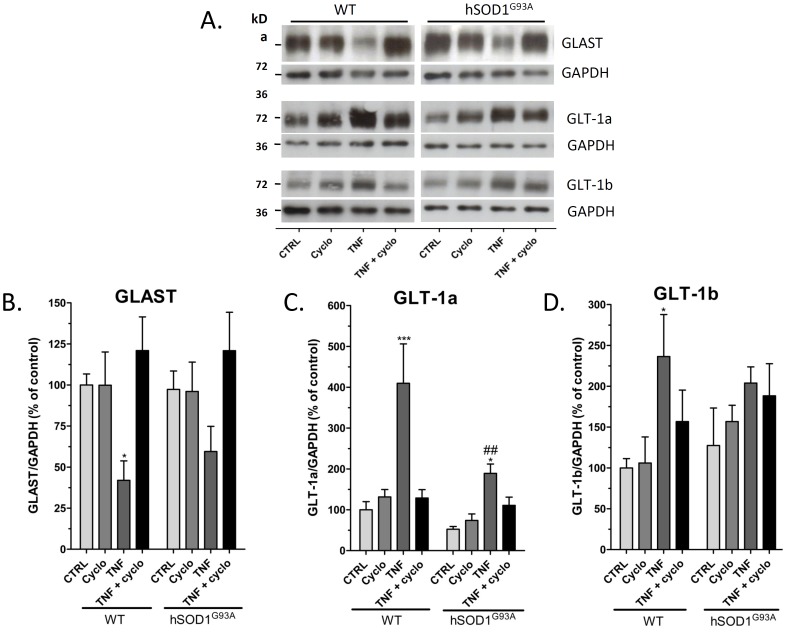
Effect of protein synthesis inhibition on the protein expression of GLAST, GLT-1a and GLT-1b on wild-type or hSOD1^G93A^ astrocytes. (A) Protein expression of GLAST, GLT-1a and GLT-1b was examined by immunoblotting in astrocytes from wild-type and hSOD1^G93A^ maintained in culture either in control conditions or treated with TNF-α (20 ng/mL) and/or using the inhibitor of protein synthesis cycloheximide (10 µg/mL) for 48 h. Immunoblots shown are representative of four independent experiments. Data obtained after densitometric analyses of GLAST (B), GLT-1a (C) and GLT-1b (D) proteins are means with SEM normalized to GAPDH and expressed in percent of the signal obtained for wild-type astrocytes cultured in control conditions. * *p*<0.05 and *** *p*<0.001 for comparison between the same genotype after a different treatment and ##*p*<0.01 for comparison between different genotypes with the same treatment (two way ANOVA followed by Bonferroni post-hoc test).

The above detailed data suggest that TNF-α could affect the GLT-1 protein turnover in wild-type and hSOD1^G93A^ astrocytes. To further characterize the influence of TNF-α on the synthesis and stability of GLT-1 in cultured cortical astrocytes, experiments were repeated using cycloheximide (10 µg/ml), an inhibitor of the protein synthesis (Fig. 3). Considering the potential toxicity of this inhibitor, cell treatments with TNF-α were limited to 48 h, which is sufficient to trigger the regulation of both GLAST and GLT-1 isoforms. Per se, cycloheximide did not induce any modifications of the GLAST, but was found to totally abolish the down-regulation observed with TNF-α (Fig. 3B), both in wild-type and hSOD1^G93A^ astrocytes. Similarly, expression of the GLT-1a and GLT-1b proteins were not significantly changed after treatment with cycloheximide in astrocytes from both genotypes, but the up-regulation of these isoforms after TNF-α treatment was also totally abolished in the presence of cycloheximide (Fig. 3C & 3D). Furthermore, it appeared that these observations are not due to a decreased expression of the TNF-α receptor 1 (TNFR1) caused by cycloheximide as this receptor expression remained unchanged by Western blotting after an 8h exposure to the drug (data not shown).

### Regulation of Glutamate Transporter Activity by TNF-α

**Figure 4:**
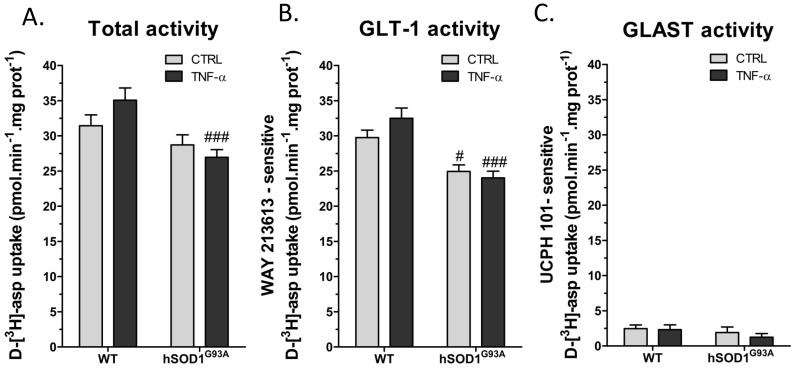
Influence of TNF-α on the activity of glutamate transporters from wild-type or hSOD1^G93A^ cortical astrocytes. Glutamate transporter activity was evaluated by measuring velocity of d-[^3^H]-aspartate uptake (50 nmol/L) in wild-type and hSOD1^G93A^ astrocytes treated or not with TNF-α (20 ng/mL) for 72 h. (A) shows total uptake while (B) and (C) illustrate GLT-1- and GLAST-dependent uptake, respectively evaluated in presence of the selective inhibitors WAY-213613 (100 µmol/L) and UCPH-101 (10 µmol/L). Shown are mean with SEM from five independent experiments realized in quintuplicate. #*p*<0.05 and ###*p*<0.001 for comparison between different genotypes with the same treatment.

The overall aspartate uptake measured on cultures from wild-type astrocytes revealed that the treatment with TNF-α caused a modest increase in the glutamate transporter activity which, however, did not reach statistical significance. The transport activity in cultures from hSOD1^G93A^ astrocytes was slightly lower as compared to wild-type, but more obvious was the absence of TNF-α influence on these cells. Thus, in TNF-α-treated cultures, the uptake measured on hSOD1^G93A^ astrocytes was significantly lower than on wild-type astrocytes (Fig. 4A). The nature of the glutamate transporters supporting this uptake and its regulation was examined using the selective pharmacological inhibitors WAY-213613 (GLT-1) and UCPH-101 (GLAST). As summarized in Fig. 4C, GLAST weakly contributed to the aspartate uptake in these conditions and most of the uptake was indeed depending on GLT-1 (Fig. 4B). Also, the GLT-1-dependent uptake was slightly increased in wild-type astrocytes treated with TNF-α while such regulation was not observed in hSOD1^G93A^ astrocytes.

## Discussion

Decreased glutamate uptake and neuroinflammation are well-documented features of several neurodegenerative disorders, including ALS [22]. While these pathological mechanisms are frequently considered independently, it is suggested that altered glutamate handling by astrocytes could result from local inflammation [15]. Indeed, regulation of glutamate transporters by pro-inflammatory cytokines was already reported in vitro and in vivo in models of nervous diseases. The present data clearly indicate that TNF-α induces a decrease in GLAST and an increase in GLT-1a and GLT-1b expression in cortical astroglial cultures. The down-regulation of GLAST expression in response to inflammation was already evidenced in astrocytes and TNF-α was identified as a key mediator of this decrease [23], [24]. A transcriptional regulation through AP-1 (activator protein 1) or CREB (cAMP response binding protein) has been proposed as a potential mechanism [25], [26]. In accordance with data from the literature indicating that GLAST is unaffected during ALS, the expression of this transporter appeared similar in astrocytes from hSOD1^G93A^ and wild-type rats. However, considering the evidence for a down-regulation of GLAST in astrocytes exposed to an inflammatory environment and the robust inflammatory response that develops in the spinal cord from ALS rats, it is surprising that the GLAST expression remains unchanged in the spinal cord during the disease progression. While the present study focused on TNF-α, other cytokines and other mechanisms of regulation could likely contribute to the progression of the disease and may have complex effects on this transporter. Also, one cannot exclude that astrocytes derived from the spinal cord could show distinct responses to inflammation. It is also worth mentioning that although GLAST mRNA is abundantly expressed, the contribution of this transporter in the total uptake activity was found to be low. This finding obtained in mature astrocytes is consistent with the common view on GLT-1 as the predominant glutamate transporter in the adult central nervous system [27].

At variance with GLAST, the influence of an inflammatory environment on GLT-1 remains controversial. In accordance with the present study, several publications report on the up-regulation of GLT-1 expression in astrocytes after exposure to pro-inflammatory mediators. We previously found that conditioned medium from LPS-activated microglia induces increased GLT-1 expression and activity [28]. However, others have described a down-regulation of GLT-1 by inflammatory mediators. For instance, a role was assigned to TNF-α during the hypoxia-mediated suppression of GLT-1 in astrocytes [29]. These inconsistencies may originate from the diversity of models, pathologies and culture methods tested (e.g. duration, concentration), which may influence the TNF-α-associated signaling pathways. Indeed, during neuroinflammation, TNF-α is described with a Janus-face [30], [31] depending on the receptor subtype (TNFR1 and TNFR2) activated by the cytokine and the associated signalling. The latter involves activation of caspases or transcription factors, including NF-κB for which different binding sites are present on the GLT-1 promoter [32]. This transcription factor activates or represses GLT-1 via two distinct pathways (N-Myc, MAPK) reflecting the complexity of the regulations in function of the model considered [33], [34].

The present study conducted in astrocyte cultures also recapitulates the different levels of expression of GLT-1a and GLT-1b, as already described in both cell and animal models of ALS [10], [11], [35]. While the distinctive role of these two isoforms remains poorly understood, the structure of GLT-1b differs from GLT-1a by the presence of a PDZ motive at the C-terminus domain. This additional motive could support the interaction with some scaffold proteins containing a PDZ domain implicated in the trafficking such as PSD-95 and PICK1 that could also ensure a chaperone role [36]–[38]. Alterations in GLT-1 protein expression are abundantly described in ALS patients and in animal models of the disease [6], [39] and a link with neuroinflammation has been proposed [15]. While the present data highlight the potential benefits operated by TNF-α against glutamate-mediated excitotoxicity, one may also predict that the up-regulation of GLT-1 preserved in hSOD1^G93A^ astrocytes would remain insufficient to compensate for the loss of glutamate uptake capacity that develops during the disease. This reinforces the concept that inflammatory reactions, although they can induce beneficial responses in neurodegenerative diseases, do not necessarily prevent excitotoxicity [40], [41]. It has even been proposed that TNF-α may induce an exacerbation of glutamate-mediated excitotoxicity in a model of ALS [42].

Prolonged exposure to cycloheximide did not change the protein levels of GLAST and GLT-1 isoforms in astrocytes, thus providing evidence for the rather long half-life of these astrocytic proteins, as previously observed [43]. Furthermore, no difference was observed between cultures from wild-type or transgenic rats, arguing against the hypothesis for an altered stability of these transporters in astrocytes expressing the fALS associated mutant form of SOD1. Nevertheless, a major finding of the present study was the loss of regulation of transporters by TNF-α when protein synthesis is inhibited, which indicates that the opposite regulations of GLAST and GLT-1 by TNF-α cannot be exclusively assigned to a down-regulation of protein synthesis or to modifications in the mRNA expression. These observations suggest that TNF-α might generate post-translational modifications or accelerate degradation of other proteins necessary for the regulation process of the transporters. We, thus, hypothesize that inhibition of the protein synthesis by cycloheximide inhibits the turnover of such partner proteins supporting the opposite regulations of GLAST and GLT-1.

In addition, the cytokine positively modulated the d-[^3^H]-aspartate uptake in wild-type astrocytes but the amplitude of the response appeared rather modest with respect to the robust increase in GLT-1 expression. Besides, the uptake activity is altered in non-treated hSOD1^G93A^ astrocytes, but the positive effect of TNF-α is clearly lost in this ALS model. The up-regulation of glutamate transporters expression in astrocytes could therefore reflect an adaptive mechanism that allows the adjustment of glutamate uptake by facilitating a more rapid turnover and trafficking of these transporters [44]. The lack of correlation between changes in the expression of glutamate transporters and the glutamate uptake/clearance has already been reported and further studies are required to explain such discrepancy.

Taken together, these data highlight an opposite regulation of GLAST and GLT-1 in cultured astrocytes when exposed to a potent pro-inflammatory stimulus. The experimental setup only allowed to study the consequences of short-term responses to inflammation in isolated astrocytes, a model that certainly differs from the sustained inflammation that can be observed in the complex environment of the nervous parenchyma. However, our data shed light on the putative contribution of neuroinflammation on the regulation of glutamate transporters that may operate in the course of neurodegenerative diseases. The intracellular pathways supporting these regulations remain largely unknown, including for individual isoforms of GLT-1. Nevertheless, the existence of distinct regulatory profiles for these isoforms further suggests the specialized role of these isoforms in physiological and pathological conditions. A better understanding of these mechanisms could provide some clues when attempting to manipulate these glutamate transporters to reduce excitotoxicity in neurodegenerative disorders.
